# Supplementary material for: Residents transitioning between hospital and care homes: protocol for codesigning a systems-level response to safety issues (SafeST study)
Source: BMJ Open. 2022 Jan 6;12(1):e050665. doi: 10.1136/bmjopen-2021-050665 (PMC8739053; doi:10.1136/bmjopen-2021-050665)
Supplement: Supplementary data [file bmjopen-2021-050665supp001.pdf]

Supplementary material

1. Search string for Grey Literature
MedNAR and Open Grey
1. (("care home*" OR "nursing home*" OR "long term care*" OR “residential*”) AND (("Medical Error*" OR "Diagnostic Error*" OR "Medication Error*" OR "drug error*" OR "Ob-server Variation" OR "Patient safety" OR incident OR incidents OR "adverse event*" OR "near miss" OR "Risk Management" OR "Safety Management" OR Accident OR Accidents OR "Accident Prevention" OR Safety OR complication) AND ("Data Collection" OR "medical record*" OR "Health Record*" OR "report* system*" OR "anonymous report*" OR "non-anonymous report*" OR "voluntary report*" OR reporting))) OR (("care home*" OR "nursing home*" OR "long term care*" OR “residential*”) AND (("Medical Error*" OR "Diagnostic Error*" OR "Medication Error*" OR "drug error*" OR "Ob-server Variation" OR "Patient safety" OR incident OR incidents OR "adverse event*" OR "near miss" OR "Risk Management" OR "Safety Management" OR Accident OR Accidents OR "Accident Prevention" OR Safety OR complication) AND ("Data Collection" OR "medical record*" OR "Health Record*" OR "report* system*" OR "anonymous report*" OR "non-anonymous report*" OR "voluntary report*" OR reporting)))

2. Search string for EBSCO (CINAHL, MEDLINE and PsycINFO)
S1 "incident report*" OR "error report*" OR "incidents report*" OR "errors report*"
S2 "care home*" OR "nursing home*" OR "long term care*" OR “residential*”
S3 S1 AND S2
S4 ("Medical Error*" OR "Diagnostic Error*" OR "Medication Error*" OR "drug error*" OR "Observer Variation" OR "Patient safety" OR incident OR incidents OR "adverse event*" OR "near miss" OR "Risk Management" OR "Safety Management" OR Accident OR Accidents OR "Accident Prevention" OR Safety OR complication) AND ("Data Collection" OR "medical record*" OR "Health Record*" OR "report* system*" OR "anonymous report*" OR "non-anonymous report*" OR "voluntary report*" OR reporting)
S5 S2 AND S4
S6 S3 OR S5
S7 Limiters Year - 2000+
S8 Limiters Language - English

3. Search string for OVID (EMBASE 1996-2021, HMIC)
S1 "incident report*" OR "error report*" OR "incidents report*" OR "errors report*"
S2 "care home*" OR "nursing home*" OR "long term care*"
S3 residential*
S4 S2 AND S3
S5 S1 AND S4
S6 "Medical Error*" OR "Diagnostic Error*" OR "Medication Error*" OR "drug error*" OR "Observer Variation" OR "Patient safety" OR incident OR incidents OR "adverse event*" OR "near miss" OR "Risk Management" OR "Safety Management" OR Accident OR Accidents OR "Accident Prevention" OR Safety OR complication
S7 "Data Collection" OR "medical record*" OR "Health Record*" OR "report* system*" OR "anonymous report*" OR "non-anonymous report*" OR "voluntary report*" OR reporting
S8 S6 AND S7
S9 S5 AND S8
S10 S5 OR S9
S11 Limiters – English Language
S12 Limiters – 2000 – 2021
S13 S10 AND S11 AND S12

4. Search string for ProQuest (ASSISA)
S1 noft ("incident report*" OR "error report*" OR "incidents report*" OR "errors report*")

S2 noft ("care home*" OR "nursing home*" OR "long term care*" OR “residential*”)
S3 S1 AND S2
S4 ("Medical Error*" OR "Diagnostic Error*" OR "Medication Error*" OR "drug error*" OR "Observer Variation" OR "Patient safety" OR incident OR incidents OR "adverse event*" OR "near miss" OR "Risk Management" OR "Safety Management" OR "Accident" OR "Accidents" OR "Accident Prevention" OR "Safety" OR "complication*") AND ("Data Collection" OR "medical record*" OR "Health Record*" OR "report* system*" OR "anonymous report*" OR "non-anonymous report*" OR "voluntary report*" OR "reporting")
S5 S2 AND S4
S6 S3 OR S5
Limiter Exclude Pre 2000
Limiter English

5. Search string for Web of Science
S1 TS= ("incident* report*" OR "error* report*")
S2 TS= ("care home*" OR "nursing home*" OR "long term care*" OR “residential*”)
S3 S1 AND S2
S4 TS= ("Medical Error*" OR "Diagnostic Error*" OR "Medication Error*" OR "drug error*" OR "Observer Variation" OR "Patient safety" OR incident OR incidents OR "adverse event*" OR "near miss" OR "Risk Management" OR "Safety Management" OR "Accident" OR "Accidents" OR "Accident Prevention" OR "Safety" OR "complication*") AND TS=("Data Collection" OR "medical record*" OR "Health Record*" OR "report* system*" OR "anonymous report*" OR "non-anonymous report*" OR "voluntary report*" OR "reporting")
S5 S4 AND S2
S6 S5 OR S3
Limiter Exclude Pre 2000
Limiter English

6. Search string for Scopus
S1 TITLE-ABS-KEY ("incident report*" OR "error report*" OR "incidents report*" OR "errors report*")
S2 TITLE-ABS-KEY ("care home*" OR "nursing home*" OR "long term care*" OR “residential*”)
S3 S1 AND S2
S4 TITLE-ABS-KEY ("Medical Error*" OR "Diagnostic Error*" OR "Medication Error*" OR "drug error*" OR "Observer Variation" OR "Patient safety" OR incident OR incidents OR "adverse event*" OR "near miss" OR "Risk Management" OR "Safety Management" OR Accident OR Accidents OR "Accident Prevention" OR Safety OR complication) AND
TITLE-ABS-KEY ("Data Collection" OR "medical record*" OR "Health Record*" OR "report* system*" OR "anonymous report*" OR "non-anonymous report*" OR "voluntary report*" OR reporting)
S5 S2 AND S4
S6 S3 OR S5
Limiter Exclude Pre 2000
Limiter English
